# Doped Halloysite Nanotubes for Use in the 3D Printing of Medical Devices

**DOI:** 10.3390/bioengineering4040096

**Published:** 2017-12-15

**Authors:** Jeffery A. Weisman, Udayabhanu Jammalamadaka, Karthik Tappa, David K. Mills

**Affiliations:** 1 Center for Biomedical Engineering and Rehabilitation Science, Ruston, LA 71270, USA; jweisman@wustl.edu (J.A.W.); ujammalamadaka@wustl.edu (U.J.); kktappa@wustl.edu (K.T.); 2 School of Biological Sciences, Louisiana Tech University, Ruston, LA 71272, USA

**Keywords:** 3D printing, drug delivery, halloysite nanotubes, nanotechnology

## Abstract

Previous studies have established halloysite nanotubes (HNTs) as viable nanocontainers capable of sustained release of a variety of antibiotics, corrosion agents, chemotherapeutics and growth factors either from their lumen or in outer surface coatings. Accordingly, halloysite nanotubes (HNTs) hold great promise as drug delivery carriers in the fields of pharmaceutical science and regenerative medicine. This study explored the potential of 3D printing drug doped HNT constructs. We used a model drug, gentamicin (GS) and polylactic acid (PLA) to fabricate GS releasing disks, beads, and pellets. Gentamicin was released from 3D printed constructs in a sustained manner and had a superior anti-bacterial growth inhibition effect that was dependent on GS doping concentration. While this study focused on a model drug, gentamicin, combination therapy is possible through the fabrication of medical devices containing HNTs doped with a suite of antibiotics or antifungals. Furthermore, tailored dosage levels, suites of antimicrobials, delivered locally would reduce the toxicity of individual agents, prevent the emergence of resistant strains, and enable the treatment of mixed infections.

## 1. Introduction

Osteomyelitis is one of the more devastating diseases of the bone, and it remains a challenge to physicians in both its diagnosis and treatment [1,2]. Bone tissue can be infected in many ways; as the result of trauma, an open wound exposure, affected adjacent soft tissue, from a peripheral vascular disease, or surgery [3,4]. Bones of the lower extremity (foot and ankle) tend to be more susceptible to infection due to thin soft tissue coverings, boney protrusions, and inadequate vascular supply [4]. Certain chronic conditions, such as diabetes, increase the risk of osteomyelitis as the incidence of foot ulceration is high in diabetic patients, and these ulcerations and subsequent infection can lead to osteomyelitis [4,5,6,7]. Age-related differences in bony anatomy and its blood supply may influence the clinical incidence of osteomyelitis [3,5]. The overall age and sex-adjusted annual incidence of osteomyelitis were 21.8 cases per 100,000 persons per year [8]. The epidemiology of the condition has changed with advances in healthcare requiring adjustments in the management of osteomyelitis [9]. The increased survival rate following a traumatic injury has seen an increase in the occurrence of posttraumatic osteomyelitis [5,10,11]. Similarly, with improved life expectancy among elderly patients, those suffering from diabetes mellitus, we are witnessing more cases of neuropathy and vascular problems with associated soft-tissue losses, bone destruction, and osteomyelitis [12,13].

Osteomyelitis is classified as acute or chronic, based on the duration of infection [4,5]. Acute myelitis occurs over a period of few days to weeks and has symptoms of localized pain, edema, and redness at the site of infection. Chronic myelitis is a prolonged recurrence of an acute case and is characterized by ischemia and bone necrosis [4]. Most of the osteomyelitis is polymicrobial [4,5]. Microbes such as Staphylococcus aureus, Escherichia coli, and Pseudomonas aeruginosa are the most robust. Staphylococcus aureus is the most common causative microorganism for osteomyelitis [14].

Infections of the bone typically begin with bacterial adhesion (or another infectious organism) to the bone matrix. The attached organism causes an inflammatory response leading to bone necrosis [9,10]. Also, inflammatory factors, such as IL-1 and TNF, trigger osteoclast activity, resulting in the demineralization of bone [13]. To treat osteomyelitis effectively, antibiotic treatment and surgical methods are used, either individually or in combination, depending on the severity of the infection [9,10,14]. The choice of antibiotic therapy depends on the source of the infection and antibiotics are administered parenterally for 5 to 6 weeks, and then a prolonged course of oral administration is recommended. When antibiotic therapy proves refractory, and a considerable amount of necrotic tissue is present, surgical intervention is necessary, with the aim to remove all the necrotic bone and infected soft tissue [13,14]. Despite surgical intervention and a sustained course of antibiotic therapy, chronic recurrence of osteomyelitis occurs in about 30% of patients within a year of primary treatment [5,15,16].

Systemic delivery of antibiotics for the treatment of osteomyelitis affects the intended site as well as unaffected tissues raising the risk of cytotoxicity, nephrotoxicity and an increase in antibiotic resistance [6,7]. Additionally, antibiotic treatment given systemically is constrained due to poor penetration of blood-borne drugs into bone tissue resulting in a significant relapse rate in cases of chronic osteomyelitis. Local antibiotic delivery systems have proven effective as they can provide long-term, high antibiotic concentrations, and along with surgical debridement can provide remediation from osteomyelitis [15,17,18,19,20]. These systems are extensively reviewed in Nandi et al., 2016 [16]. Several non-biodegradable poly(methyl methacrylate) (PMMA)-based drug delivery systems have been developed for the treatment of chronic osteomyelitis and are considered the gold standard in antibiotic treatment [18,19]. Antibiotic-impregnated PMMA implants have numerous advantages over systemic therapy in the treatment of osteomyelitis. These include ease of placement, several antibiotic choices, decreased systemic toxicity, reduced hospital stays and patient cost. While effective, the disadvantages of currently used implants (e.g., PMMA (beads, nails, spacers)) include low biocompatibility, show poor antibiotic release profiles, continued antibiotics release for undefined periods, require a second and costly surgery for their removal, and produce cytotoxic effects due to thermal damage during PMMA polymerization [17,18,19,20,21].

Recently, a new class of biodegradable materials has been developed that may prove more successful in the prevention, recurrence, and complications arising from systemic therapy. These are osteoconductive bioceramics, such as calcium sulfate, tricalcium phosphate or hydroxyapatite, impregnated with antimicrobial agents, developed specifically for the local management of osteomyelitis [22,23,24]. They are placed within the resected area, and the antibiotics elute from the implants for an extended period [16]. They are also space filling, offer a wider selection of antibiotics, and require only one surgery [21,25,26,27,28]. Besides bioceramics, silicate, [29,30] bioglasses, [31] and polymers including poly(d/l-lactide) [32,33] are also under investigation. While showing much promise, these systems have some issues such as processability, a burst release of antibiotics, cytotoxicity of degradation products, none have gone through extensive clinical trials, and few have received FDA approval [33].

It is clinically accepted that direct treatment, without unnecessary and widespread harmful effects, is highly desired. 3D printing is rapidly enabling on-demand and patient-specific antibiotic treatments and has the potential to address many of the disadvantages of current antibiotic carrier systems [34,35]. Its major advantages of accuracy, speed, and ability to create complex and intricate biomedical devices is well suited towards this goal [36]. Furthermore, many studies have shown its tunability enabling customization for a specific application or a patient with a unique medical situation [37,38,39]. The ability to provide customized treatments that insert a drug-doped Implants in or near the diseased organ or tissue that provides sustained release can provide the benefits of high antibiotic therapy and low systemic toxicity.

Since 2012, Halloysite nanotubes (HNTs) have attracted significant attention as a drug nanocontainer and nanocarrier [40,41]. HNTs belong to the kaolin group of minerals and is structured as a circumferential-layered aluminosilicate with a hollow tubular lumen [42,43]. It has an inner diameter of 10–30 nm and an outer diameter of 50–70 nm, while its length varies in the range of 0.5–1.5 μm [42,43]. One of its key features is the differential charge on its surfaces as the inner surface is positively charged while the outer surface is negatively charged, offering many opportunities for loading biologically active agents into the HNT lumen and within coatings applied to the HNT surface, followed by their sustained release [44,45,46]. The role of HNTs as a nanocontainer and nanocarrier was discussed in detail by Abdullayev and Lvov (2013) [43]. Its use as a nanofiller for the modification of nanocomposite materials has long been known, and HNTs have been used to increase surface area, impart high surface reactivity, improve mechanical strength with a relatively low cost. To achieve increases in the toughness, mechanical strength, and thermal stability, HNTs have incorporated into a variety of polymers. Examples include: poly(butylene succinate), [47] polyamide 12, [48] styrene-butadiene, [49] epoxy, [50] poly(methyl-methacrylate) [46] and chitosan [51]. In several previous studies using electrospinning, HNTs were successfully incorporated into electrospun scaffolds and were effective in releasing a suite of bioactive factors [52]. HNTs added to calcium phosphate cement were shown to produce an osteoinductive effect on pre-osteoblasts [53]. Recently, Kelnar et al., 2016 showed that PLA fibrils formation combined with the reinforcement of components by HNTs increased PLA crystallinity leading to a more biocompatible and biodegradable material with enhanced mechanical performance [54].

Accordingly, we hypothesized that HNTs, combined with biodegradable thermoplastics, can be used to produce high-resolution medical devices and scaffolding materials with complex geometries and tailored drug release profiles. This study was designed to test this prediction. HNTs were doped with the antibiotic, gentamicin (GS), and used to create customized filaments for use in 3D printing of biomedical construct including beads, disks, and filaments. Our results indicate that the released gentamicin retains its thermal stability and bacterial growth inhibition activity. Inhibition of bacterial growth was achieved using a 7.5 wt % GS/HNT 3D printed construct.

## 2. Materials and Methods

### 2.1. Materials 

The 3D printing set-up consisted of an ExtrusionBot extruder purchased from ExtrusionBot, LLC (Phoenix, AZ, USA) and a MakerBot Replicator 2X 3D printer purchased from MakerBot (Brooklyn, NY, USA). The clear polylactic acid (PLA) pellets used for the printing media were obtained from NatureWorks, LLC (Minnetonka, MN, USA). PLA was chosen since it is FDA approved, biodegradable and economical. Gentamicin sulfate (GS) used in this study was purchased from Sigma-Aldrich (St. Louis, MO, USA). KJLC 705 silicone oil used for coating the pellets before extrusion was purchased from Kurt J. Lesker Company (Jefferson Hills, PA, USA). Bacterial cultures were derived from *E. coli* vitroids (Sigma-Aldrich, St. Louis, MO, USA). For drug detection 2-hydroxyethyl mercaptan and ophthalaldehyde (OPTA) were purchased from Sigma-Aldrich, St. Louis, MO, USA. HNT nanotubes with a length of 30–70 nm and a pore volume of 1.26–1.34 mL/g were purchased in powder form from Sigma-Aldrich, St. Louis, MO, USA.

### 2.2. Methods

#### 2.2.1. Vacuum Loading of Gentamicin

For drug loading, GS was vacuum-loaded into HNTs. HNTs (250 mg/mL.) were mixed with a 2 mL GS solution (250 mg/mL). The mixed suspension was placed in a vacuum overnight. An additional 2 mL of DI water was added and the process repeated two times. These HNTs were centrifuged to separate from the solution and were air dried. 

#### 2.2.2. Preparation of PLA Filaments

**Figure 1:**
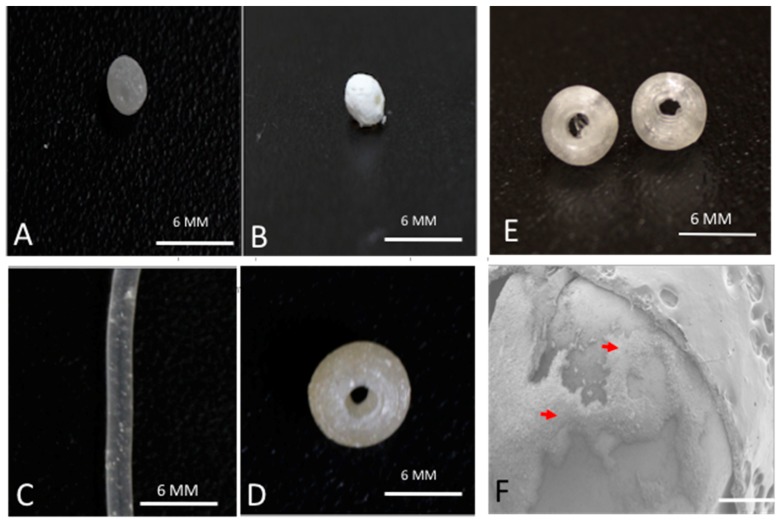
Coated pellets, filaments and beads. (**A**) 1.5 wt % gentamicin (GS) loaded halloysite nanotubes (HNTs), coated on polylactic acid (PLA) pellet; (**B**) 10 wt % gentamicin loaded HNTs, coated on PLA pellet; (**C**) 7.5 wt % GS-HNT 3D printed PLA filament; (**D**) 7.5 wt % GS-HNT 3D printed PLA bead; (**E**) 3D printed PLA control bead; (**F**) SEM of GS-HNT coated pellet, red arrows indicate GS coating, bar = 5 mm.

We developed a new method that enables an even dispersion of drugs on the surface of commercially available PLA pellets before the extrusion process (Figure 1) [37,38]. To surface coat pellets, KJL 705 silicone oil was chosen because of its thermal stability at extrusion temperatures of 170–180 °C. In this study, we use 20 g batch of pellets, to which was added 15 μL of silicone oil and then vortexed to make sure all the pellets were evenly and completely coated. Once vortexed, the pellets were transferred to another container to avoid loss of drug powder due to sticking to the surface of the oil-coated mixing container. After switching containers, a calculated amount of GS-HNT in powdered form was added and vortexed again. 1.5 and 7.5 wt % GS-HNT-PLA pellets were made as shown in Figure 1. 

#### 2.2.3. PLA Filament Extrusion

An ExtrusionBot filament extruder was used for extruding filaments of required diameter. It consisted of a vertical column with a feed hopper leading directly into a melt chamber housing an auger within a heated pipe. What the metal die at the extrusion point was 1.75 mm diameter. A control panel on the front modifies the temperature as needed. The typical extrusion temperature of PLA pellets is around 170 °C depending on ambient temperature and humidity conditions. Each 20 g of GS coated PLA pellets were extruded at 170 °C maintaining the out coming filament diameter as 1.75 mm. We also tried extruding below 170 °C, but that slowed down the extrusion speed and resulted in a thicker filament that could not be used in 3D printing. At higher temperatures, PLA melts completely and flows through the metal die causing thermal runaway. Since this equipment works on piston based auger system, back pressure was necessary. Small batches of pellets could not provide sufficient pressure and stayed inside the extruder for a longer time-periods resulting in an excessive heating and thin filament extrusion. For PLA, 20 g batches extruding at 170 °C were optimum conditions for getting filament of 1.75 mm diameter. Since the filament extrusion process involves high temperatures, GS was used in this research due to its high thermal stability properties. In previous studies, thermal denaturation studies were conducted with GS and showed that its bioactivity was unaffected by heating at temperatures up to 220 °C, far above the extrusion temperature, and on par with the printing temperature [37]. 

#### 2.2.4. 3D Printing

The SolidWorks computer software modeling program (Dassault Systèmes SolidWorks Corporation, Waltham, MA, USA) was used to design the 3D print files. CAD designs were drawn and visualized in SolidWorks for Windows and transferred to a 3D printable format using the MakerWare software supplied with the Replicator 2X. The resulting generated. STL file was used to dictate the construct dimensions to the printer through MakerWare. The default standard settings; Extrusion temperature at 215 °C, layer height of 0.2 mm, travelling speed at 150 mm/s was used for 3D printing the HNT-GS constructs. All constructs were printed at 10% infill ratio with diamond shaped infill pattern.

#### 2.2.5. Gentamicin Release Profile

To determine the elution profile for our model drug gentamicin sulfate (GS), we used a NANODROP 2000 UV-Visible spectrophotometer from Thermo Scientific (Waltham, MA, USA). GS-loaded and extruded filaments (1 cm length), beads, and catheters were tested. Simulated Body Fluid (SBF) was prepared by mixing NaCl (7.996 g), NaHCO_3_ (0.350 g), KCl (0.224 g), K_2_HPO_4_·3H_2_O (0.228 g), MgCl_2_·6H_2_O (0.305 g), HCL (1 M, 40 mL), CaCl_2_ (0.287 g), Na_2_SO_4_ (0.071 g), and (CH_2_OH)_3_CNH_2_ (6.057 g). This SBF was used to collect the samples from the constructs periodically. The time intervals for collecting samples were taken at selected intervals over a 48-h period. Since GS could not be directly detected, indirect determination using OPTA reagent was done. Equal volumes of collected sample, isopropyl alcohol, and OPTA reagent were added, and this mixture was analyzed using spectrophotometer at 330 nm. SBF was used as a blank for all these tests. Measured quantities of drugs were used to draw standard graphs and from these absorbance values, the amount of drug eluted in the samples was back calculated. 

#### 2.2.6. Bacterial Cultures

Printed constructs were tested for inhibition of bacterial growth using Muller-Hinton agar plates. In addition to above groups, negative controls were also tested for antibiotic activity. A set of positive controls was used and included the antibiotic powder of respective categories. *E. coli* vitroids were used to raise bacterial colonies, and one colony was picked to make standard Muller-Hinton agar plates. 3D printed GS-HNT-PLA pellets, disks and filaments and loaded with different percent concentrations of GS were tested. Undoped 3D printed PLA, and HNT-PLA pellets, disks and filaments were used as negative controls. All cultures were incubated for 24 h at 37 °C in an incubator and zones of inhibition were measured at three different points, including the sample at the center, using digital calipers and were averaged. 

#### 2.2.7. Liquid Broth Cultures

Mueller Hinton culture broths were inoculated with 50 μL of bacterial cultures and 3D printed GS-HNT-PLA pellets, disks, and filaments and loaded with different percent concentrations of GS were tested. These cultures were incubated for 24 h at 37 °C on a rocker. Absorbance values of these cultures were recorded using a GENESYS20-Thermospectronic spectrometer at 600 nm with clear broth as background. Triplicates of each group were tested and compared with controls.

#### 2.2.8. Statistical Analysis

A one-way ANOVA test was performed at a significance level of 0.05. The standard deviation of the means (*n* = #/group) was calculated and used as error bars in each graph. 

## 3. Results

### 3.1. Fabrication of Antibacterial Disks and Filaments

**Figure 2:**
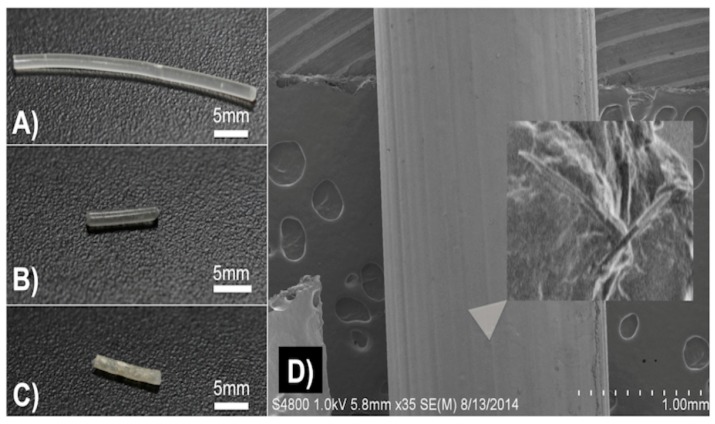
(**A**) 7.5 wt % HNT loaded with gentamicin PLA filament; (**B**) 1.5 wt % HNT loaded with gentamicin PLA filament; (**C**) 10 wt % HNT PLA filament; (**D**) SEM 1.5 wt % HNT loaded with gentamicin filament. Insert magnifies the filament surface in D to show GS flakes.

We used a method previously described to prepare the gentamicin-coated beads (+/− HNTS) for use in the filament extrusion process (Figure 1A,B). Fabrication of filaments doped with HNTs and gentamicin were extruded into filaments with no problems experienced during the extrusion process (Figure 1C,D). There was no clogging of the extruder or print heads and fabrication of HNT doped and drug doped HNTs into beads, disks and filaments occurred with high fidelity (Figure 2A–C).

### 3.2. SEM Analysis

SEM was used to document that GS-HNT coatings were applied to the pellets as shown in Figure 1F and that they appeared as a uniform coating over the PLA bead. It should be noted that before SEM, the samples needed to be cleaned with compressed air which removed some of the powdered coatings so some coating was assumed lost during SEM processing. A darkening in color was observed with the GS and HNT addition to PLA in printed constructs (Figure 2A–C). With increasing HNT percent addition printed constructs also slightly increased in surface roughness, and GS flakes were visible (Figure 2D).

### 3.3. Growth Inhibition Studies

**Figure 3:**
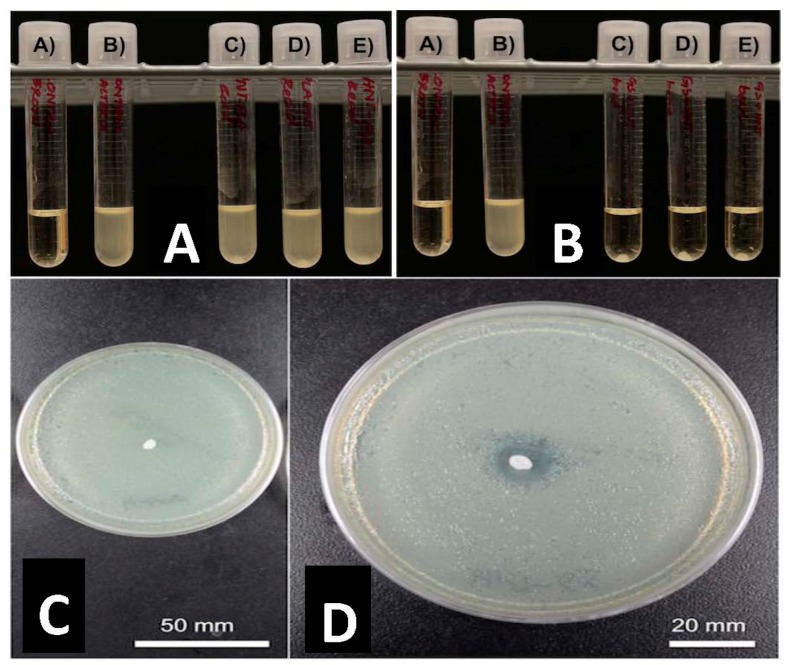
PLA pellets growth inhibition studies. (**A**): (A) Sterile broth; (B) *E. coli* inoculated broth’ (C–E) 10 wt % HNT-coated PLA pellets in broth culture. (**B**): (A) sterile broth; (B) *E. coli* inoculated broth’ (C–E) 1.5 wt % gentamicin loaded HNT-coated PLA pellets in broth culture. (**C**): 10 wt % HNT-coated PLA pellet on Mueller Hinton plate. (**D**): 1.5 wt % gentamicin loaded HNT-coated PLA pellets on Mueller Hinton plate.

**Figure 4:**
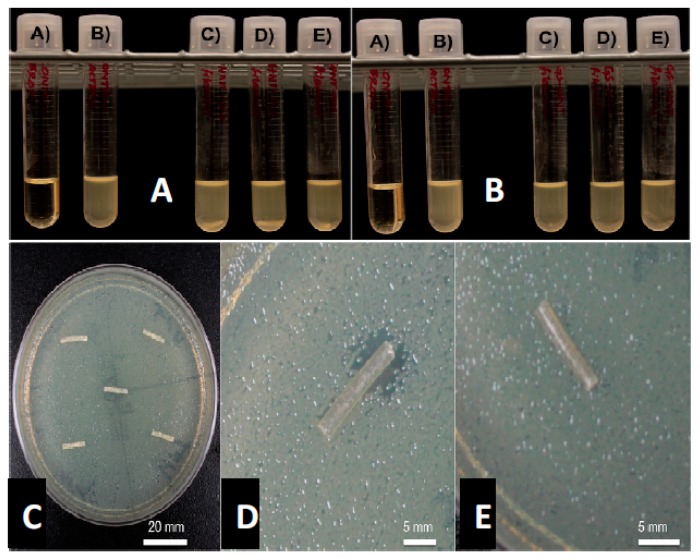
(**A**): (A) Sterile broth; (B) *E. coli* inoculated broth; (C–E) 10 wt % HNT doped PLA filaments. (**B**): (A) Sterile broth; (B) *E. coli* inoculated broth; (C–E) 1.5 wt % gentamicin loaded HNT doped PLA filaments. (**C**): 10 wt % HNT-PLA filament; (**D**,**E**): 1.5 wt % HNT loaded with gentamicin PLA filament.

**Figure 5:**
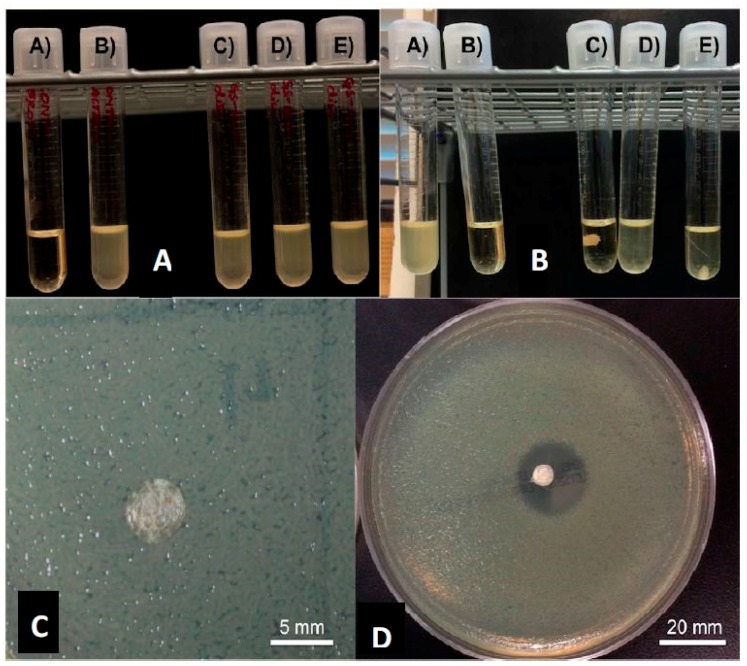
(**A**): (A) Sterile broth, (B) *E. coli* inoculated broth (C–E) 1.5 wt % GS-loaded HNT-PLA discs. (**B**): (A) *E. coli* inoculated broth; (B) sterile broth; (C–E) 7.5 wt % GS-loaded HNT-PLA discs. (**C**): 1.5 wt % HNT loaded with gentamicin disc; (**D**): 7.5 wt % GS-loaded HNT loaded disc.

**Figure 6:**
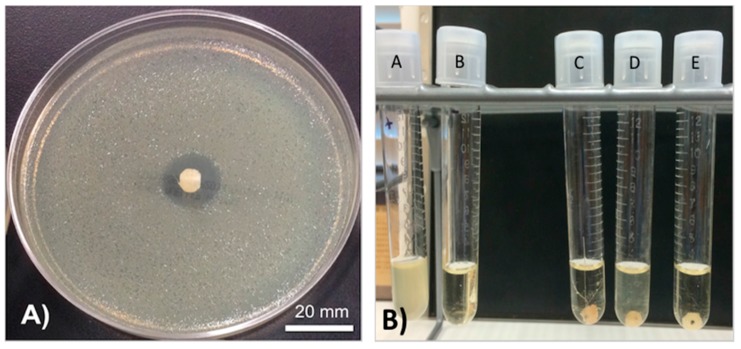
(**A**): 7.5 wt % GS-loaded HNT-PLA 3D-fabricated bead; (**B**): (A) = *E. coli* inoculated broth, (B) = sterile broth; (C–E) = 7.5 wt % GS-loaded HNT-PLA fabricated bead.

GS was released from HNTs and all 3D printed disks (Figure 3C,D). In Figure 3, a 1.5% GS-HNT doped pellet shows a small zone of growth inhibition. In broth cultures, 1.5% GS-HNT doped pellet was effectively at reducing bacterial growth as compared to controls (Figure 3A,B). In contrast, a 1.5% GS-HNT doped filament had a much-reduced zone of inhibition as compared to the pellet and controls (Figure 4C,D). Broth cultures also showed a limited effectiveness at reducing bacterial growth (Figure 4A,B). In contrast, a 7.5 wt % HNT loaded gentamicin disc showed a much larger show of inhibition (Figure 5) an observation confirmed by broth cultures of a similar GS doped disks. Figure 6 compares growth inhibition of a 1.5% and 7.5% loaded gentamicin disc, the higher concentration of GS/HNTs produced a much more effective growth inhibition response. 

**Figure 7:**
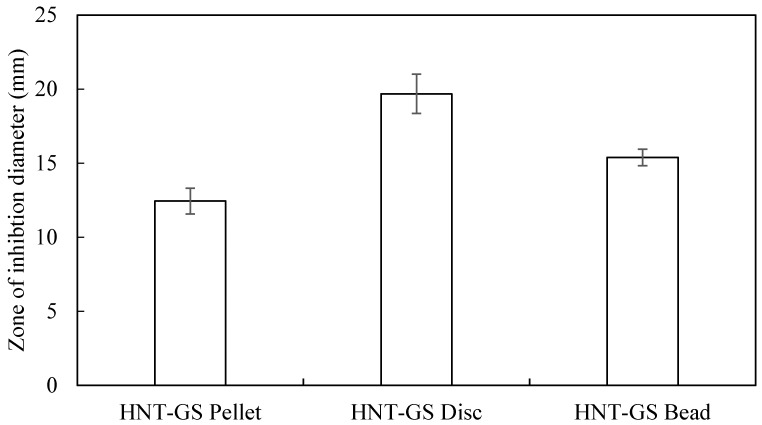
Zone of inhibition diameters for different HNT-GS constructs against *E. coli* (mean ± SD, *n* = 3).

The graph in Figure 7 shows the diameters of inhibition zones for different HNT constructs. Due to the low surface area, there were no kill zones for the extruded filaments. Interestingly we observed there was no inhibition zone surrounding the filaments in the agar plates probably due to small surface area. Since 3D printed HNT-GS discs have more diameter (surface area), inhibition diameters were higher than the pellets and 3D printed beads.

**Figure 8:**
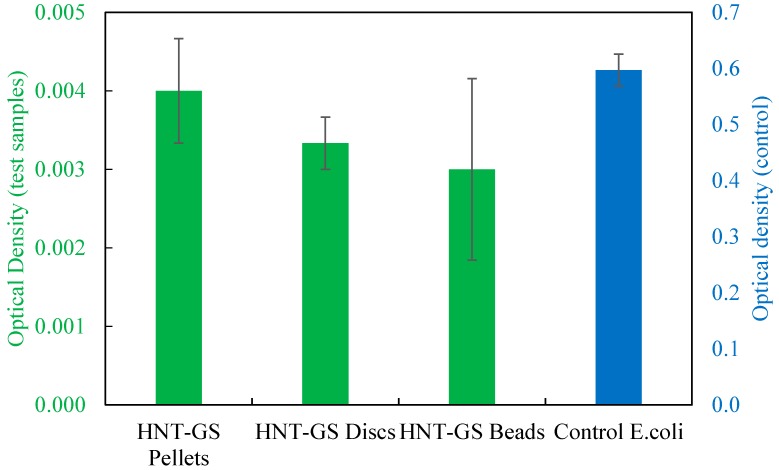
Optical densities of different HNT-GS constructs against *E. coli* (mean ± SD, *n* = 3) measured at 600 nm.

The absorbance values of the LB agar broth with HNT-GS scaffolds against *E. coli* cultures were measured at 600 nm as shown in Figure 8. The optical density of the broth culture tube is directly proportional to its turbidity. More the bacterial growth higher is the optical density. So, the control cultures had extremely higher optical density values than antibiotic constructs. To accommodate both the control and test optical density values, the *Y*-axis on the left (green) was ruled with lower absorbance values for the test samples containing HNT-GS specimens and *Y*-axis on the right (blue) was calibrated with high absorbance value for the control broth containing *E. coli* alone. Among all the HNT-GS constructs, pellets showed highest optical density values when compared to 3D printed discs and beads. This may be due to gradual release of GS from the HNTs embedded within the 3D printed constructs when compared to the quick surface release from HNT pellets. Since the polymer used is biodegradable, the anti-infective effect would be sustained and extended. 

### 3.4. Drug Release Profile

**Figure 9:**
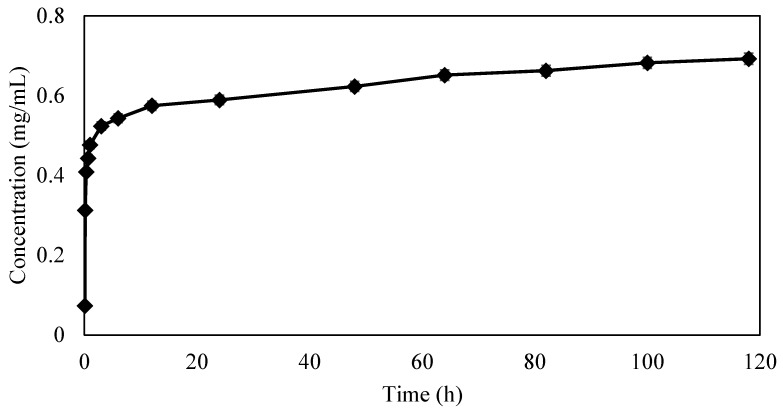
Cumulative concentration (mg/mL) of GS released from HNTs.

The graph in Figure 9 shows the cumulative concentration of GS released from HNTs over a period of 120 h. There was an initial outburst of drug followed by a steady extended release. The amount of GS eluted from the 3D printed constructs were below the sensitivity range of spectrophotometer and could not be detected. 

## 4. Discussion

Systemic delivery of antibiotics affects the intended site as well as unaffected tissues, and for some patients, raises the risks of increased patient sensitivity, cytotoxicity, nephrotoxicity and the potential for an increase in antibiotic resistance. The local administration of anti-infective agents can deliver higher antibiotic concentrations to infected sites and without the harmful side effects associated with the parenteral route [5,6,8,9]. Poly(methyl methacrylate) (PMMA) is the most commonly used non-biodegradable carrier material, and it is used to deliver antibiotics in the form of antibiotic-doped beads, nails, or spacers [55,56,57]. The devices provide high concentrations of broad-spectrum antibiotics to sites of infection; in particular, to bone sites that are difficult to reach by systemically delivered antibiotics [58,59]. Antibiotic-doped PMMA does have a few major disadvantages including high exothermic temperatures generated during PMMA polymerization, limitations on the types of antibiotics that may be employed, insufficient antibiotic release rates, and the need for a second surgery to remove implanted PMMA constructs [59,60].

The use of antibiotic-laden bioabsorbable or biodegradable material has been under intense investigation in recent years [59,60]. These materials have been shown promise as an alternative treatment for osteomyelitis as they can deliver an extended release of antibiotics without the toxicity associated with PMMA. Silicate-based systems [60] and bioglasses [61] have been studied for the treatment of chronic osteomyelitis. However, osteoconductive bioceramics (calcium sulfate, tricalcium phosphate or hydroxyapatite) or bioceramic composites infused with antibiotics combine antibiotic therapy with osteoconductive capabilities [62,63,64]. Their major advantage is they strongly bond with bone, fill bone voids, and will convert to hydroxyapatite that promotes osteogenesis at the defect site [65,66]. Like PMMA, they have a few disadvantages and include lack of solid clinical data regarding conditions for use, optimum dosages, elution properties, and pharmacokinetics are still poorly defined [60]. However, the consensus view that high drug concentrations provided locally and for a sustained period is highly desired [58,59].

As 3D printing enables on-demand and patient-specific antibiotic treatments it has the potential to address many of the disadvantages of current antibiotic carrier systems. Furthermore, many studies have shown its ability to provide novel solutions for a specific clinical application or customized for a patient with a unique medical situation [37,38,39]. The ability to provide customized treatments (combinatorial therapy) that insert a drug-doped implant in or near the diseased organ or tissue providing sustained release can provide the benefits of high antibiotic therapy and low systemic toxicity. Furthermore, 3D printing permits the design of drug release system dependent on the carrier and a patent’s specific clinical situation. Accordingly, we studied the potential of using a powder-based FDM method to 3D print GS doped HNT-PLA composites as an implantable drug delivery system. Powder-based FDM techniques have been used in many applications as it allows unlimited scaffold architectures and the precision to create high-resolution models [65,66]. 3D printed bone scaffolds have been developed that support cell adhesion, mimic the extracellular matrix of bone, and allow complex drug release geometries [39,67,68]. Powder-based 3D printing has also been used to formulate orally disintegrating acetaminophen tablets and oral dosage forms of chlorpheniramine maleate again, with a complex release profile [69,70]. 

We critically assessed our FDM printed HNT-PLA composites’ drug releasing capabilities and anti-bacterial effects. There was sustained release of GS from 3D printed scaffolds, and 3D printed constructs with a higher percent concentration of GS-HNT were more effective in inhibiting bacterial growth. A significant research effort had been directed towards the use of PLA and clay nanocomposites in applications ranging from drug delivery, regenerative medicine, and 3D printing [71,72,73]. PLA/HNTs nanocomposites have been fabricated using several methods including injection molding [74], melt mixing [75], and solvent casting [76] for various applications. De Silva investigated the influence of processing techniques on the resultant material properties of PLA/HNT nanocomposites [77]. In almost all cases, additives such as quaternary ammonium salt [16] or silane [15], were used to enhance the composites elastic and impact properties or toughness. These previous studies, the extensive studies by Weisman (2014) and our own, suggest that fabrication methods of PLA/HNTs nanocomposites can influence their drug-releasing and physicochemical properties. 

Humans are physiologically and biologically unique. Accordingly, 3D printed medical devices, tissues and organs must become personalized to each patient’s unique anatomy and physiology or customized to fit a specific condition or illness [78]. A key goal in this field is to combine the key features of 3D printing (accuracy, speed, tunability) into a system that enables on-demand and patient-specific antibiotic treatments. Current antibiotic delivery systems, such as PMMA bead strings, are severely limited in their drug release capabilities, ability to deliver a multi-spectrum drug regimen, or a combination of drugs [79]. Furthermore, the non-porous nature of PMMA bone cement allows very little antibiotics and [80] current commercially available cement formulations release less than 3% of the total gentamicin sulfate during the first 10 days, with at least 70% of that released within the first 24 h [81,82].

**Figure 10:**
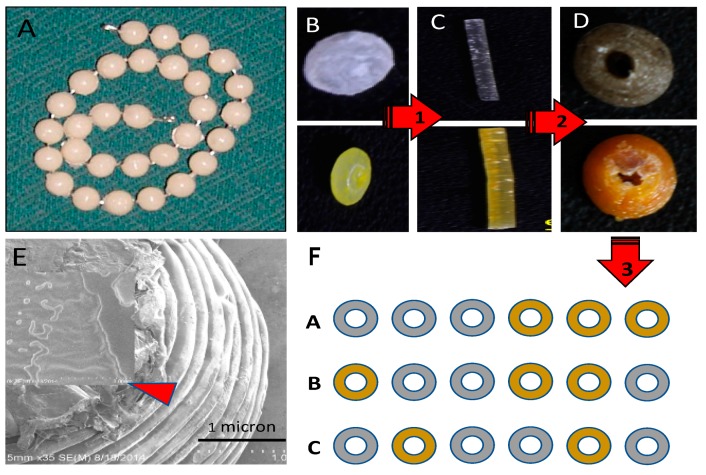
(**A**) Commercially available antibiotic-laden PMMA bead string. (**B**) Drug doped PLA pellets, gentamicin pellet (upper figure) and methotrexate pellet (lower figure); (**C**) Extruded drug-doped-filaments. Gentamicin-doped filament (upper figure) and methotrexate-doped filament (lower figure). (**D**) 3D printed drug doped beads. These can then be assembled into implantable bead strings. Gentamicin bead (upper figure) and methotrexate bead (lower figure). (**E**) SEM of a 3D printed bead showing later-by-layer assembly with gentamicin crystals embedded within the assembled layers. (red arrow, insert). (**F**) Potential antibiotic designs demonstrating the potential for 3D printing diverse sets of bioresorbable bead strings. Arrows (1–3) indicates the direction of the fabrication process (pellet>>>extruded filament>>>3D printed bead).

Our device can be fabricated to specifically fit each patient’s condition and site of infection (Figure 10). Drug-doped HNTs can be printed into a single strand of biodegradable beads or disks (PLA or PCL) with customized features including a suite of antibiotics, different dosage levels, drug combinations such as anti-microbials and chemotherapeutics without disadvantages with systemic delivery systems [63,64]. Its use would result in a lower serum antibiotic concentration than that is usually associated with systemic administration, thereby reducing toxicity-related side-effects. Considering the most commonly described microbes causing chronic osteomyelitis, the most widely acceptable antimicrobial agents in local delivery systems are amino glycosides and to a lesser extent various beta-lactam agents and quinolones [16,20,21]. However, a combination therapy of antibiotics would reduce the toxicity of individual agents, prevent the emergence of resistance and enable the treatment of mixed infections [16]. A dual-or multi-functional 3D printed bioresorbable implant has significant potential as new therapeutic approach for infected bone defects such as periapical bone lesions or contaminated bone fractures. Since the 3D printed antimicrobial devices are also biodegradable there is no need for secondary surgery as they are gradually replaced by ingrowing tissue, and may even support new bone growth. Additionally, antibiotic released during degradation should increase drug efficacy compared to non-biodegradable carriers. 

## 5. Conclusions

We have demonstrated that it is possible to print antibiotics into implant devices and influence their release profile thus supporting the concept that a customized and patient-specific antibiotic delivery system is possible through 3D printing. GS loaded HNT constructs were successfully fabricated using 3D printing technology. SEM images showed the presence of HNTs on the surface of the polymers. Bioactivity of the fabricated constructs were successfully tested using zone of inhibition and broth culture methods. Clear demarcating kill zones for the *E. coli* cultures were seen for 3D printed GS-HNT discs and beads.

HNTs can be doped with a suite of antibiotics, different dosage levels, different anti-microbials and customized for a specific patent. Since the 3D printed antimicrobial devices are also biodegradable there is no need for secondary surgery as they are gradually replaced by ingrowing tissue, and may even support new bone growth. Additionally, antibiotic release during degradation should increase drug efficacy compared to non-biodegradable carriers. 

Since the fused deposition modeling process involves elevated temperatures to sinter the polymer layers, this technology is limited only to thermally stable bioactive substances. In this research, we used PLA polymer which sinters at 215 °C and could successfully fabricate biodegradable implant material without losing its bioactive property. Biopolymers (or combination of polymers) with lower sintering temperatures should be more researched and can potentially be an ideal material for loading drugs and fabricating bioactive implants.
